# Proposal and Verification of the Theory of Layer-by-Layer Elimination of Biofilm in *Listeria monocytogenes*

**DOI:** 10.3390/foods12071361

**Published:** 2023-03-23

**Authors:** Jialin He, Xiangyu Gao, Hanbing Huang, Jianxiong Hao

**Affiliations:** College of Food Science and Biology, Hebei University of Science and Technology, No. 26 Yuxiang Street, Shijiazhuang 050018, China

**Keywords:** *Listeria monocytogenes*, biofilms, electrolysed water, confocal laser scanning microscopy, layer-by-layer elimination

## Abstract

Biofilms are microbial communities that represent a high abundance of microbial life forms on Earth. Within biofilms, structural changes during clearance processes occur in three spatial and temporal dimensions; therefore, microscopy and quantitative image analysis are essential in elucidating their function. Here, we present confocal laser scanning microscopy (CLSM) in conjunction with ISA-2 software analysis for the automated and high-throughput quantification, analysis, and visualisation of biofilm interiors and overall biofilm properties in three spatial and temporal dimensions. This paper discusses the removal process of *Listeria monocytogenes* (LM) biofilms using slightly acidic electrolytic water, non-electrolytic hypochlorite water, and alternating the use of strongly acidic and strongly alkaline electrolytic water. The results show that the biofilm gradually thins and gutters from the initial viscous dense and thick morphology under the action of either biocide. This process is consistent with first-level kinetics. After CLSM filming to observe the biofilm structure, analysis software was used to process and quantify the biovolume, average biofilm thickness, biofilm roughness and other indicators; fluorescence enzyme markers were used to verify the remaining amount of extracellular nucleic acid. In this study, we proposed and validated the theory of layer-by-layer elimination of LM biofilm.

## 1. Introduction

*Listeria monocytogenes* (LM) is a Gram-positive bacillus that can grow in extreme environments. It is one of the world’s four most recognised foodborne pathogens and can produce a haemolytic exotoxin [1]. It spreads easily and can contaminate a variety of foods, such as meat products, aquatic products, and plant foods. LM is not only present in food items but also in the pipeline of food processing and transportation [2]. Listeriosis, caused by LM, is the third most lethal of all foodborne illnesses [3]. Infection with this bacterium is usually associated with fever and muscle aches, along with diarrhoea or other symptoms of gastroenteritis, sepsis, and meningitis, which are the most common symptoms in the elderly and immunocompromised individuals [4,5]; infection with LM can also lead to the miscarriage of pregnant women and the death of newborns [6].

Biofilms (BFs) are defined as “aggregates of microorganisms in which cells are embedded in self-generated extracellular polymeric substances (EPSs) attached to the surface” [7]. These EPSs are mainly polysaccharides, proteins, lipids, and nucleic acids, which provide mechanical stability to the biofilm and form a three-dimensional polymer network that interconnects BF cells [8]. Bacterial BFs are an emerging life form based on the EPS matrix. They represent a protective model for bacterial cells that can survive in harsher environments and show greater resistance to antimicrobials than planktonic bacteria [9]. The biofilm is divided from the outside to the inside into a biofilm layer, a connecting layer, a conditioned layer, and a matrix layer [10]. Bacteria form BFs in four stages: adhesion, aggregation, maturation, and shedding, which are complex and dynamic processes [11]. The biofilm can effectively protect the bacteria in the biofilm, making the bacteria more resistant to unfavourable environments (such as hyperacidity and alkalinity, high temperature, and high osmotic pressure) and to drugs by helping the bacteria better adapt to the surrounding environment [12,13,14]. Therefore, it is important to choose an appropriate and efficient sterilisation method to effectively kill bacterial biofilms and explore the mechanism of biofilm removal.

Quantifying surface biofilm formation is challenging because traditional microbial methods, which often rely on manual counting, are susceptible to human error. In contrast, the direct imaging of biofilms using microscopic techniques provides information about their structural characteristics, which in turn can determine whether interventions are successful in disrupting biofilm formation. Confocal laser scanning microscopy (CLSM) selectively excites fluorescent signals from different planes in the sample, acquiring images point-by-point under local laser excitation at specific wavelengths and allowing for 3D visualisation of the biofilm structure by excluding signals from adjacent planes. The second benefit of CLSM is the versatility offered by the fluorescent dyes added to the sample, which allows for the acquisition of additional information such as the presence of extracellular DNA, extracellular polysaccharides, and biofilm viability. Among the available fluorescent staining methods, live/dead staining is a traditional method for evaluating biofilm formation in microorganisms with a wide range of applications, including in oral, skeletal, and intestinal microorganisms [15,16]. In combination with live/dead staining, CLSM can be used to quantify biofilm viability on transparent and opaque surfaces. Additionally, in combination with extracellular polymer dyes, the dynamics of BFs during formation and clearance can be observed.

Validation experiments have shown that automated analysis is an improvement over traditional microbial analysis and is a reliable measure of biomass and cell viability [17]. CLSM and image analysis software can be used to quantitatively assess the structural features of biofilms at a lower cost than scanning electron microscopy (SEM) and transmission electron microscopy (TEM). The 3D spatial structure within the biofilm can be obtained by fluorescent staining in combination with CSLM, calculated in conjunction with image analysis software, and quantified for structural parameters, such as average thickness and biovolume [18]. Understanding the 3D structure of EPSs is important because they can promote antimicrobial resistance in biofilms by hindering the transport of certain antibiotics [19]. Disruption of the biofilm structure results in cell exposure and enhances the action of biocides, which is a potential solution for the prevention of bacterial re-resurgence on the surface of objects; therefore, it is an important aspect to consider. In this study, CLSM was combined with the analysis software, ISA-2 [20], to analyse the resulting 3D images. ISA-2 contains ISA for 2D and 3D parameters [21].

In the last decade, various strategies based on chemical, physical, and biological agents for inactivating harmful bacteria in food have emerged with great advantages, such as ozone [22], electrolytic oxidation of water [23], high-pressure carbon dioxide [24], and phages [25]. However, the use of these methods in the food industry still has some limitations. Electrolysed water (EW) is a general term for acidic and alkaline electrolytic water obtained through the electrolysis of diluted salt or hydrochloric acid water using an electrolytic water generation device. This results in changes in the pH, oxidation-reduction potential (ORP), effective chlorine concentration (ACC), and a series of other indicators of the water system; good acidity, alkalinity, and redox properties make it easy to prepare and environmentally friendly [26]. As a new type of bactericidal agent, acidic electrolytic water (AEW) has been recognised for its strong bactericidal efficacy. It was used as a food additive for food disinfection in Japan in 2002 [27]. Alkaline electrolysed water (ALEW) generally has a strong cleaning function. It is an efficient and inexpensive agent for stripping organic matter and other substances [28]. Slightly acidic electrolytic water (SAEW) has been used in sanitation and cleaning systems in the food industry for many years. It has shown antimicrobial activity against a variety of microorganisms in a relatively short period of time [29,30,31].

Most of the current studies on LM BFs remain at the method level, with less research on the removal mechanism, and even less on the physical level changes when LM BF is removed. Accordingly, the objective of this study was as follows: (i) use SAEW, NEHW, and ALEW/AEW as bactericide to understand in detail the mode of action of biocides, especially their interaction with the EPS matrix and other BF components; (ii) this study considers the biofilm removal process as a dynamic process, which has an important applications in biofilm removal from surfaces. Therefore, this paper proposes the theory of “biofilm elimination layer-by-layer”.

## 2. Materials and Methods

### 2.1. Materials

The strain used in this study was LM ATCC19114 (Beijing Solarbio Science & Technology Co., Ltd., Beijing, China), stored at −80 °C. The nucleic acid standards, calf thymus DNA and yeast RNA were purchased from Beijing Solarbio Science & Technology Co., Ltd. and stored at −20 °C. The fluorescent dyes that bind tightly to biofilms were acridine orange (AO), propidium iodide (PI), and Syto^®^9 (LIVE/DEAD^®^ Biofilm Viability Kit), which were purchased from Beijing Solarbio Science & Technology Co., Ltd. and stored at 4 °C.

### 2.2. Preparation of Treatment Solutions

Preparation of SAEW (pH: 5.6, ACC: 50 mg/L): Electrolytes (such as NaCl and HCl) were added to the water. The laboratory-built diaphragm-free electrolyser was used to prepare SAEW.

Preparation of non-electrolytic hypochlorite water (NEHW, pH: 5.6, ACC: 50 mg/L): NaOCl was added to the water, and the pH was adjusted to obtain the required non-electrolytic hypochlorite solution.

Preparation of AEW (pH: 2.3, ACC: 50 mg/L)/ALEW (pH: 10.5, ACC: 10 mg/L): NaCl was added to water for electrolysis through an ion exchange membrane. Strongly acidic electrolytic water was generated on the anode side, and strongly alkaline electrolytic water was generated on the cathode side.

The main active substance in AEW is chlorine gas, which reacts with water to produce HCl and hypochlorous acid (HClO). The effective chlorine in SAEW and NEHW is HClO, which further decomposes to form neo-ecological oxygen [O]. The new ecological oxygen species undergo extremely strong oxidation, denaturing the proteins of bacteria and viruses, thus causing the death of pathogenic microorganisms. HClO in the bactericidal and viricidal processes can act on the cell wall and virus shell. Additionally, the small uncharged molecules of HClO can penetrate the bacterial cell (virus) and proteins, nucleic acids, and enzymes (such as those that catalyse oxidation reactions) which lead to the death of pathogenic microorganisms.

### 2.3. Preparation of BF

LM was incubated in plates for 24 h at 37 °C, and single colonies were inoculated into 200 mL of TSB liquid medium and incubated at 160 rpm at 37 °C for 24 h. The strain was activated and prepared in a viable bacterial solution with a viable count of approximately 10^8^–10^9^ cfu/mL and set aside. Subsequently, 1 mL of bacterial solution was placed on each glass slide on the culture dish, and 25 mL of TSB-Ye liquid medium was added around the glass slides. Glass slides were placed in a constant temperature incubator, and the culture medium was changed every other day for 5 days until the BF reached the mature stage.

### 2.4. Use of Treatment Solutions

Removal of LM biofilm by bactericides: Slides covered with BF were treated with SAEW and NEHW for 2, 4, 6, 8, and 10 min; strongly acidic electrolytic water and strongly alkaline electrolytic water were used alternately with the following treatments: A_1_S_4_, A_2_S_8_, S_3_A_1_S_1_, and S_6_A_2_S_2_ (where S stands for strongly acidic electrolytic water, A stands for strongly alkaline electrolytic water, and the number represents the action time). A 0 min treatment group using only Phosphate-Buffered Saline (PBS) without biocides was set up as a blank control group. Calculation of germicidal rate: After treatment with bactericides, the slides were placed in a culture dish with 5 mL of saline, and the biological membranes on both sides of the slides were scraped off with a cell spatula and collected in a 10 mL centrifuge tube. The treated samples were diluted in a gradient, coated and inoculated into solid medium, and incubated on nutrient agar plates for 24 h. The results are expressed as log_10_cfu/mL. Observation of biofilm morphology: After treatment with bactericides, the washing of LM using PBS was followed by dye staining.

### 2.5. Staining of Extracellular Products

The BFs were stained using the method previously described by Peng et al. (2020); the dye information is shown in Table 1. Then, 50 µL of dye was added to each BF, which was followed by staining with AO for 15 min, PI, and Syto^®^9 for 5 min. AO can fluoresce in different colours depending on the amount of DNA and RNA binding in the cell, with less green fluorescence with DNA binding and more orange fluorescence with RNA binding. PI is a DNA-binding dye that produces red fluorescence. It cannot penetrate the membrane of living cells and can only stain dead cells. Therefore, when observed under a fluorescence microscope, normal cells cannot be coloured, early apoptotic cells show faint red light, late apoptotic cells show enhanced red light, and necrotic cells show strong red fluorescence. Syto^®^9 infects all bacteria regardless of membrane integrity. However, PI only enters bacteria with damaged membranes, resulting in reduced Syto^®^9 fluorescence. Therefore, using the optimised mixture, dead bacteria turned red and live bacteria turned green.

### 2.6. CLSM Observation

Mature BFs covered with glass slides were subjected to sterilisation treatment as described in the “Use of treatment solutions” section. However, instead of collecting the samples, they were washed with PBS and stained using the stains shown in Table 1. The BFs were washed with PBS to remove excess dye, air-dried, and scanned using a Leica confocal microscope at 512 × 512 pixels. Followed by the visualisation of LM BFs using a confocal microscope (Leica TCS SP8) imaging application, the filter set and laser settings utilised the Alexa 488 fluorophore. Three evenly distributed fields of view were randomly selected for each sample, and each treatment was repeated in triplicate.

### 2.7. ISA-2 Analysis

CLSM images were analysed using ISA-2 software (provided by Prof. Haluk Beyenal, Montana State University, Bozeman, MT, USA) to determine biofilm structural parameters, such as BV, MT, porosity (P), and biofilm roughness (BFR). This was performed to quantify the BF structure for statistical analysis [32]. BV corresponds to the volume occupied by the bacteria, MT indicates the spatial size of the BF, BFR is a measure of FB thickness variation, and P represents the tightness of the inter-BF association, with a larger P indicating a more dispersed BF.

### 2.8. Extraction of Extracellular Substances

The biocide-treated slides were placed in a Petri dish filled with 5 mL of saline. The BFs on the front and back of the slides were collected in a 10 mL centrifuge tube with a cell spatula, fixed to 6 mL, and then treated with constant temperature shaking (150 r/min, 1 h, room temperature), water bath (1 h, 60 °C), followed by centrifugation (9000 rpm, 20 min, 4 °C), and filtering through a 0.45 µm filter membrane to obtain the supernatant (i.e., the extracellular polymer) [33].

### 2.9. Determination of Fluorescence Intensity

Detection of DNA and RNA changes: The supernatant was injected into a 96-well plate. Three parallel experiments were performed for each treatment, and the average value was recorded. AO was added for staining, and the reaction was protected from light for 15 min. The relative fluorescence intensity was measured using a multi-mode microplate reader (SpectraMax^®^M2, Molecular Devices Instruments Co., Shanghai, China).

### 2.10. Degradation Kinetic Model

A degradation kinetic model was constructed for the remaining DNA and RNA contents after SAEW and NEHW treatment of BFs with treatment times of 0–10 min and 2 min intervals, respectively. The principles are as follows: assuming that the number of reaction stages is zero, one, or two, the remaining extracellular nucleic acid concentration *ρ* is linearly related to time *t*, the logarithm of the remaining extracellular nucleic acid concentration is ln(*ρ*/*ρ*_0_) to time t, and the inverse of the remaining extracellular nucleic acid concentration is 1/*ρ* to time *t*. The degradation kinetic model was determined by the highest value of the corresponding linear regression coefficients, and the models are shown in Equations (1)–(3).

(1) $$\rho -\rho_{0}=-k_{0}t$$

(2) $$ln(\rho /\rho_{t})=-k_{1}t$$

(3) $$1/\rho -1/\rho_{0}=-k_{2}t$$

### 2.11. Statistical Analysis

Each treatment was repeated thrice. For each treatment, data from independent replicate trials were pooled, and the means and standard deviations were calculated. All data were analysed using the Duncana multiple range test (SPSS16.0 for Windows, SPSS Inc., Chicago, IL, USA). Differences between treatments were established at a significance level of *p* < 0.05. Charts were drawn using Origin software (OriginPro 8, OriginLab, Northampton, MA, USA).

## 3. Results

### 3.1. Germicidal Rate

**Figure 1:**
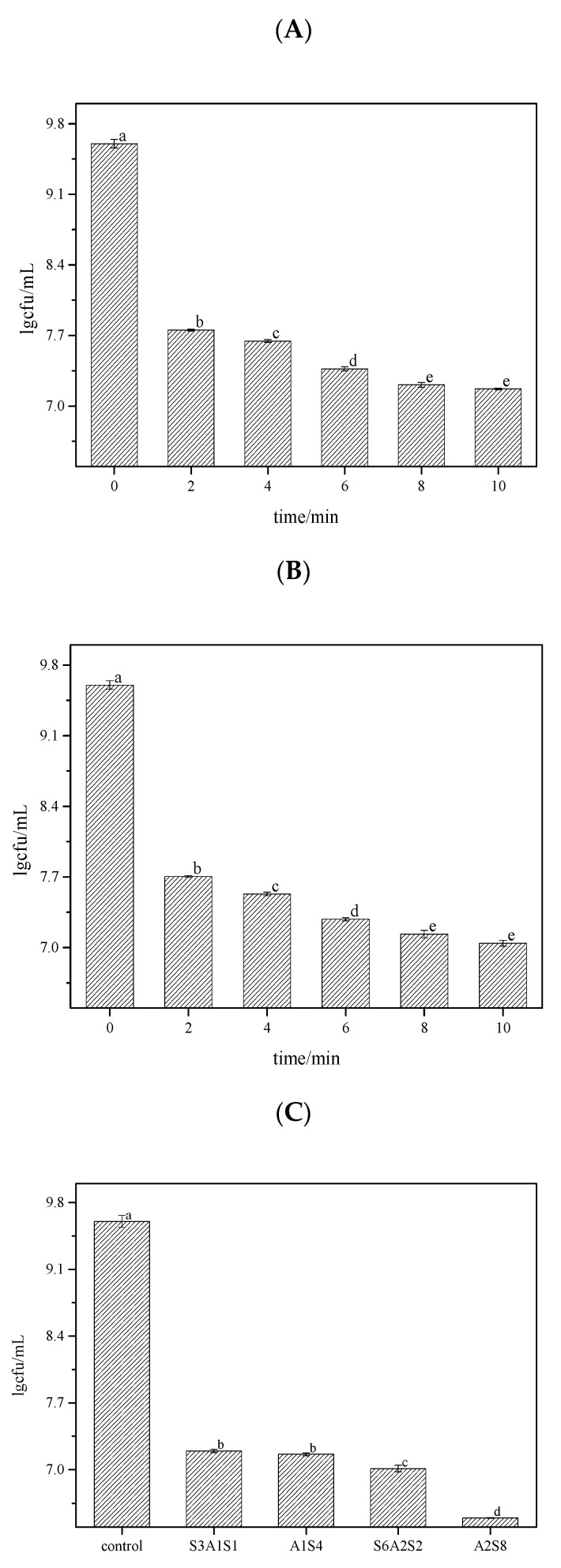
The bactericidal rate of each treatment was determined by the coated plate method after treating LM biofilm with three groups of bactericides for different times. The different letters indicate significant differences (*p* < 0.05). Data were expressed by mean measurements ± standard deviation (SD), and all treatments and determinations were performed in triplicate. (**A**) is the bactericidal rate of SAEW. (**B**) is the bactericidal rate of NEHW. (**C**) is the bactericidal rate of AEW and ALEW. SAEW was the abbreviation of slightly acidic electrolytic water, and NEHW was the abbreviation of non-electrolytic hypochlorite water. In A_1_S_4_, A_2_S_8_, S_3_A_1_S_1_, and S_6_A_2_S_2_ treatment, S stands for strongly acidic electrolytic water, A stands for strongly alkaline electrolytic water, and the number represents the action time.

After treating LM BFs with three groups of bactericides for different times, the bactericidal rate of each treatment was determined by the coated plate method. The results are shown in Figure 1.

Relative to the control, lg9.60, the amount of LM decreased continuously with an increase in SAEW treatment time. Moreover, the amount of LM decreased by lg2.39. The bactericidal rate reached 99.58% after 8 min of treatment (*p* < 0.05) and 99.63% after 10 min of treatment. However, there was no significant difference between the 8- and 10-min treatment times. The amount of LM decreased continuously with increasing NEHW treatment time relative to the control. Additionally, the amount of LM decreased by lg2.45 with a treatment time of 8 min, and the bactericidal rate reached 99.66% after 8 min of treatment (*p* < 0.05) and 99.72% after 10 min of treatment. When AEW and ALEW were alternately used, 5 min of treatment can drop LM by lg2.4; the sterilisation rates were 99.61% and 99.64%, respectively. The best effect was achieved under a 10-min treatment with an initial treatment with AEW for 2 min and ALEW treatment for 8 min; the sterilisation rate was 99.92%.

SAEW and NEHW treatment for 2 min can have a significant sterilisation effect. Treatment for 8 and 10 min, respectively, showed no significant difference. Treatment with ALEW-AEW is better than treatment with AEW-ALEW-AEW, which is probably because ALEW can reduce the adhesion of BF and because this experiment uses elution sterilisation. After reducing the adhesion, it is easier for the BF to fall off from the carrier, which reduces the protection of the bacterium by EPS. AEW can then be used to increase the removal sterilisation effect. The initial usage of AEW stimulates a stress reaction in BFs, making sterilisation difficult; thus, even after three alternating effects, its effect is still not as good as the effect of using ALEW first followed by AEW.

### 3.2. CLSM Observation of Staining Results with ISA-2 Software Analysis

The use of CLSM allows the visualisation of general BF structures, where bright heavily stained cells are thought to be clustered together in tower-shaped bacterial aggregates, while areas that do not contain cells are identified as black background. In addition, CLSM can be used to depict three-dimensional images (the following images show the front view on the left and cross-section on the right). Exploring the structural changes in the BF as sterilisation time changes allows us to understand the dynamics of the LM BF and the mechanism of bactericidal action, with the aim of achieving better sterilisation results.

### 3.3. Dyeing with PI and Syto^®^9 after BF Treatment with SAEW

**Figure 2:**
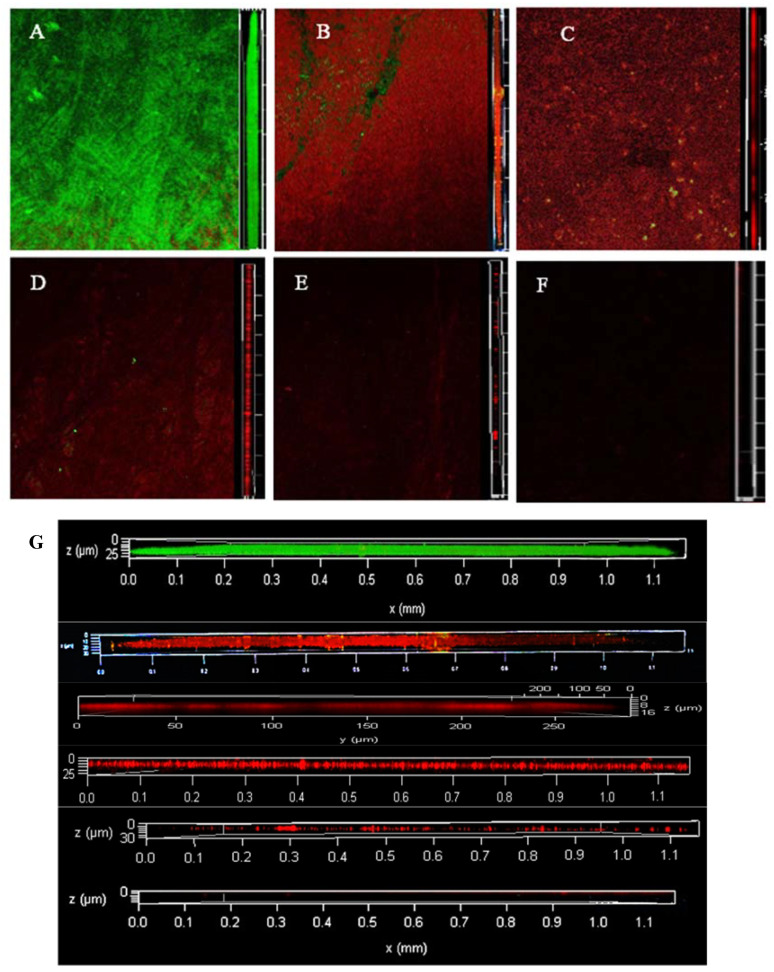
The changes of viable and dead colonies in LM biofilm after SAEW treatment observed by CLSM; each sample randomly selects three visual fields. Images include front and side views of the biofilm under CLSM. Scale bar—100 µm. (**A**–**F**) represent the dead or alive status of the remaining bacteria after 0, 2, 4, 6, 8 and 10 min of treatment, respectively. (**G**) is an enlarged version of all the images on the right and is also a right-hand view of the biofilm, from top to bottom, for SAEW treatments 0, 2, 4, 6, 8 and 10 min respectively. SAEW was the abbreviation of slightly acidic electrolytic water. Green—live cells, red—dead cells.

Figure 2 shows the change in dead and live colonies in the BF of LM observed via CLSM. At 0 min, all live bacteria almost completely covered the abiotic surface with a very dense and flat homogeneous cell layer; very few areas showed dead bacteria. At 2 min, the BF started to fragment, the structure started to degrade, and there was a large area of bacterial death since CLSM imaging was performed from bottom to top and the surface-dead bacteria were not protected by EPS; therefore, some of them were exposed to the surface showing a green colour. At 4 min, LM is no longer intact, dissipating into smaller BF structures, with individual colonies appearing. Additionally, the surface of the object is partially exposed. At 6 min, the BF was no longer a continuous and tightly packed form, showing low biomass and an increasing number of individual colonies. At 8 min, almost all cells were distributed as single cells, cell aggregation was very small, and the BF biomass and the number of both live and dead bacteria decreased compared with shorter treatment time. At 10 min, no fluorescence signal was observed, and almost no LM was attached to the object surface.

**Figure 3:**
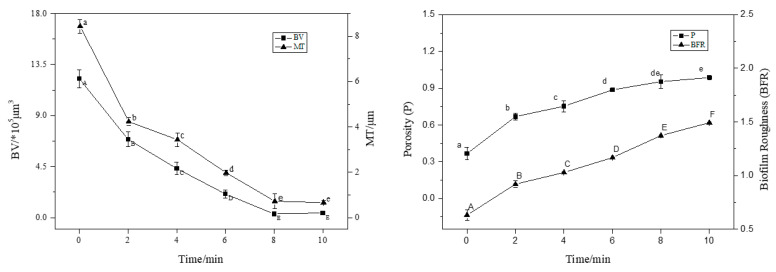
The CLSM images analysed by ISA-2 software to determine the biovolume (BV), mean thickness (MT), porosity (P) and biofilm roughness (BFR) of the biofilm. Data were expressed by mean measurements ± standard deviation (SD), and all treatments and determinations were performed in triplicate. The different letters indicate significant differences (*p* < 0.05).

CLSM images were analysed using ISA-2 software(provided by Prof. Haluk Beyenal, Montana State University, USA) to determine BV, MT, P, and BFR of the BF. The results are shown in Figure 3.

With an increase in treatment time (2–8 min), the BV and MT decreased significantly (*p* < 0.05), and the BF thickness decreased by 50% compared to the control (0 min) after 4 min of treatment. At 8 min of treatment, the average thickness was 0.2 μm and the average volume was 1.0 × 10^5^ μm^3^; the average thickness and average volume of BF were extremely small, and the BV and MT decreased in rate. This trend was consistent with the sterilisation rate. Interestingly, the P and roughness coefficient increased with increasing action time, and the BFR of the BF was the largest at 10 min, indicating that the BF surface was not smooth and formed an uneven surface structure.

### 3.4. Dyeing with PI and Syto^®^9 after BF Treatment with NEHW

**Figure 4:**
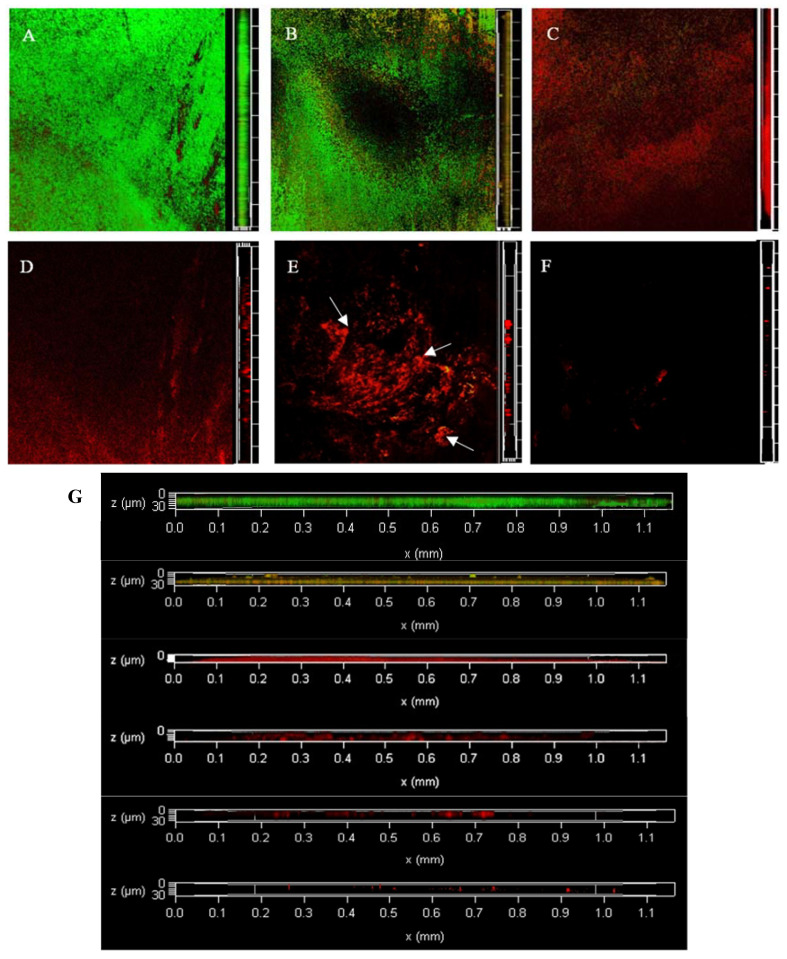
The changes of viable and dead colonies in LM biofilm after NEHW treatment observed by CLSM; each sample randomly selects three visual fields. Images include front and side views of the biofilm under CLSM. Scale bar—100 µm. (**A**–**F**) represent the dead or alive status of the remaining bacteria after 0, 2, 4, 6, 8 and 10 min of treatment, respectively. (**G**) is an enlarged version of all the images on the right and is also a right-hand view of the biofilm, from top to bottom, for NEHW treatments 0, 2, 4, 6, 8 and 10 min respectively. NEHW was the abbreviation of non-electrolytic hypochlorite water. Green—live cells, red—dead cells. The arrows point to areas of denser biofilm.

Figure 4 shows the changes in LM colony viability observed using CLSM. At 0 min, live bacteria almost completely covered the surface, with dense and coarse *Listeria* spp.; only a few dead bacteria were observed. At 2 min, the LM began to break, the structure began to degrade, and the surface of the object was partially exposed. The scavenging effect of NEHW on the BF was slightly better than SAEW. The BF thickness decreased, the dead bacteria were eluted, and a large area of green living bacteria were exposed to the surface of the object. At 4 min, the BF was no longer complete and dissipated into a smaller *Listeria* structure. A single colony appeared, and the exposed *Listeria* died over a large area. At 6 min, the BF structure was destroyed, and the biological activity decreased. At 8 min, the structure of the BF similar to the cell tower was obvious (as shown by the arrow). The rest of the BFs that were not clustered together were eluted, and the number of both live and dead bacteria was greatly reduced. At 10 min, there was no BF observed and almost no LM was attached to the surface of the object.

**Figure 5:**
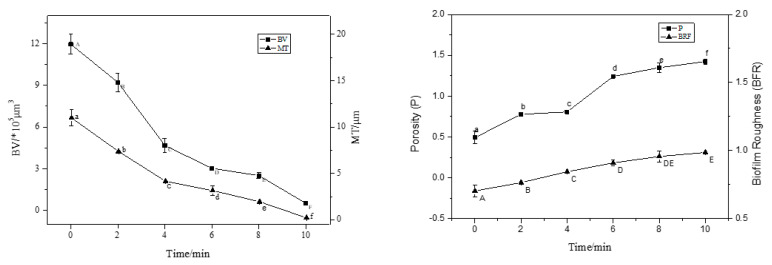
The CLSM images analysed by ISA-2 software to determine the biovolume (BV), mean thickness (MT), porosity (P) and biofilm roughness (BFR) of the biofilm. Data were expressed by mean measurements ± standard deviation (SD), and all treatments and determinations were performed in triplicate. The different letters indicate significant differences (*p* < 0.05).

CLSM images were analysed using ISA-2 software to determine the BF BV and average thickness. Figure 5 shows that BV and MT decreased significantly with increasing treatment time (*p* < 0.05). The structure of the BF became increasingly dispersed with increasing treatment time, and the average thickness and average volume decreased by 98% and 95% compared to the control group (0 min), respectively, relative to the initial values. This clearly shows the removal effect of NEHW on the BF. It is important to clarify that the removal effect of biocides on BFs is influenced by the maturity of the BF itself; that is, the thicker the BF, the larger the volume of the organism and the more bacteria it contains. Thus, a longer treatment time is required to achieve the same removal effect.

### 3.5. Dyeing with PI and Syto^®^9 after BF Treatment with AEW and ALEW

**Figure 6:**
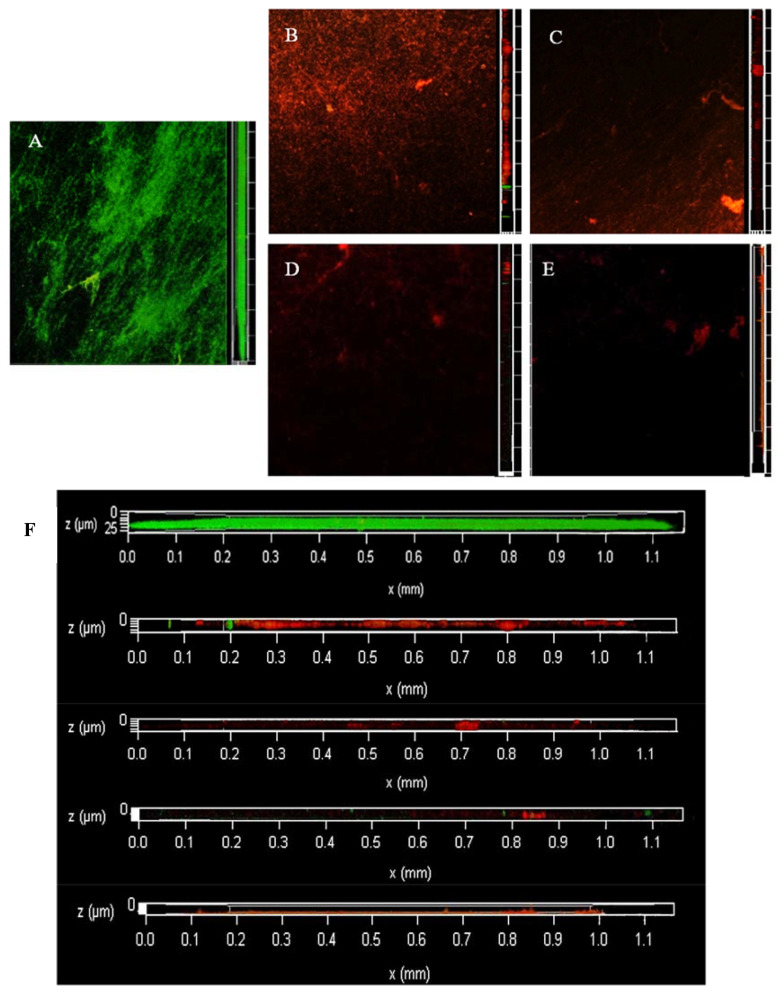
The changes of viable and dead colonies in LM biofilm after AEW and ALEW treatment observed by CLSM; each sample randomly selects three visual fields. Images include front and side views of the biofilm under CLSM. Scale bar—100 µm. (**A**) is control, (**B**) is S_3_A_1_S_1_ treatment, (**C**) is A_1_S_4_ treatment, (**D**) is S_6_A_2_S_2_ treatment, and (**E**) is A_2_S_8_ treatment. (**F**) is an enlarged version of all the images on the right and is also a right-hand view of the biofilm, from top to bottom, for AEW and NEHW treatments 0, 2, 4, 6, 8 and 10 min respectively. Green—live cells, red—dead cells. AEW was acidic electrolytic water, and ALEW was alkaline electrolysed water.

Figure 6 shows changes in dead and live LM colonies observed by CLSM. The untreated control BF showed a dense and interlinked green colour; that is, the living bacteria formed an interactive net-like BF on the cell surface through extracellular polymer aggregation, showing a complex tissue structure. Only a few areas showed yellow colour overlapping with the green colour presented by dead and living bacteria. The S_3_AS_1_ treatment showed degradation of the reticular structure. The surface of the object was partially exposed, most of the bacteria lost their vitality, and a single colony appeared. The BF in the S_6_A_2_S_2_ treatment was no longer intact, the BF consisted of only sparse aggregates and a small amount of extracellular polymeric material, and LM died on a large scale. The bactericidal effect was significant, and most of the BF was eluted; however, some remained under CLSM.

**Figure 7:**
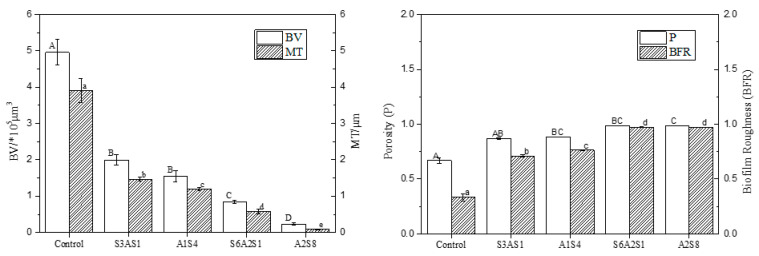
The CLSM images analysed by ISA-2 software to determine the biovolume (BV), mean thickness (MT), porosity (P) and biofilm roughness (BFR) of the biofilm. Data were expressed by mean measurements ± standard deviation (SD) and all treatments and determinations were performed in triplicate. The different letters indicate significant differences (*p* < 0.05).

As illustrated in Figure 7, the BV and average thickness decreased with an increase in treatment time. The best removal effect was in the A_2_S_8_ treatment, where the average thickness of BF was 0.09 μm and the average volume was 0.2 × 10^5^ μm^3^ after treatment. The BFR and P increased to 0.97 and 0.99, and the MT and BV of the BF were extremely small.

### 3.6. Dyeing with AO after BF Treatment with SAEW

**Figure 8:**
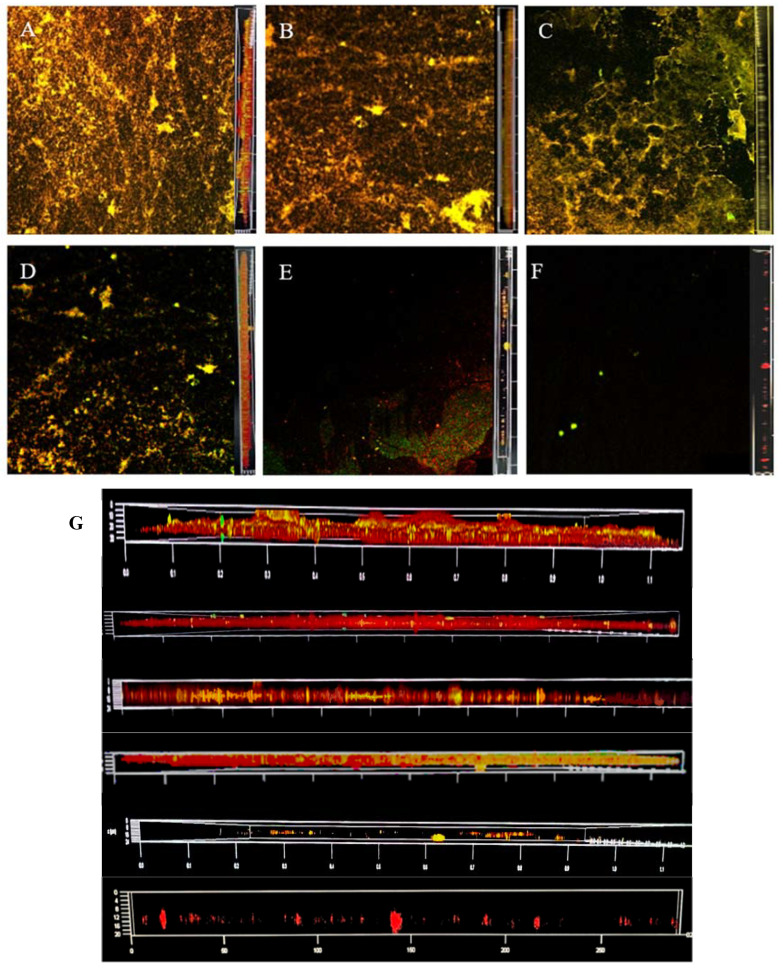
The changes of eDNA and eRNA in LM biofilm after SAEW treatment observed by CLSM; each sample randomly selects three visual fields. Images include front and side views of the biofilm under CLSM. Scale bar—100 µm. (**A**–**F**) represent treatment 0, 2, 4, 6, 8, 10 min, respectively. (**G**) is an enlarged version of all the images on the right and is also a right-hand view of the biofilm, from top to bottom, for SAEW treatments 0, 2, 4, 6, 8 and 10 min respectively. SAEW was the abbreviation of slightly acidic electrolytic water.

**Figure 9:**
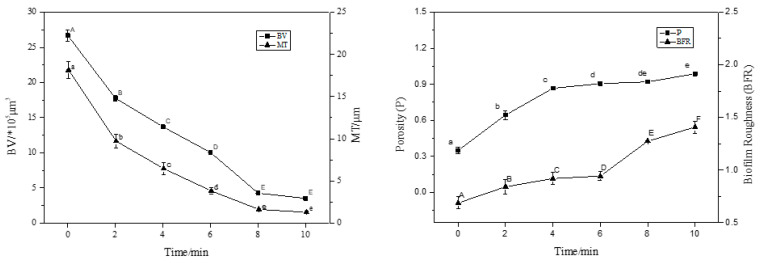
The CLSM images analysed by ISA-2 software to determine the biovolume (BV), mean thickness (MT), porosity (P) and biofilm roughness (BFR) of the biofilm. Data were expressed by mean measurements ± standard deviation (SD) and all treatments and determinations were performed in triplicate. The different letters indicate significant differences (*p* < 0.05).

Changes in the three-dimensional structure of LM during the action of SAEW were examined by CLSM and analysed using ISA-2 software. Figure 8 and Figure 9 show that in the control (0 min), the eDNA and eRNA were distributed in a high-density mesh-like viscous BF with channel-like structures passing through them. This allows for the transportation of oxygen and nutrients to the bacterium. The changes from treatment at 2–8 min showed that the eDNA and eRNA content gradually decreased, the BF thickness decreased to 1.62 μm at 8 min and the BV decreased to 4.25 × 10^5^ μm^3^, with no significant difference from 1.28 μm and 3.52 × 10^5^ μm^3^ at 10 min of treatment. There was a very small amount of eDNA and eRNA remaining at 10 min of treatment, and the P and BFR increased to 0.98 and 1.41; this greatly reduced the BF coverage and BF activity.

### 3.7. Dyeing with AO after BF Treatment with NEHW

**Figure 10:**
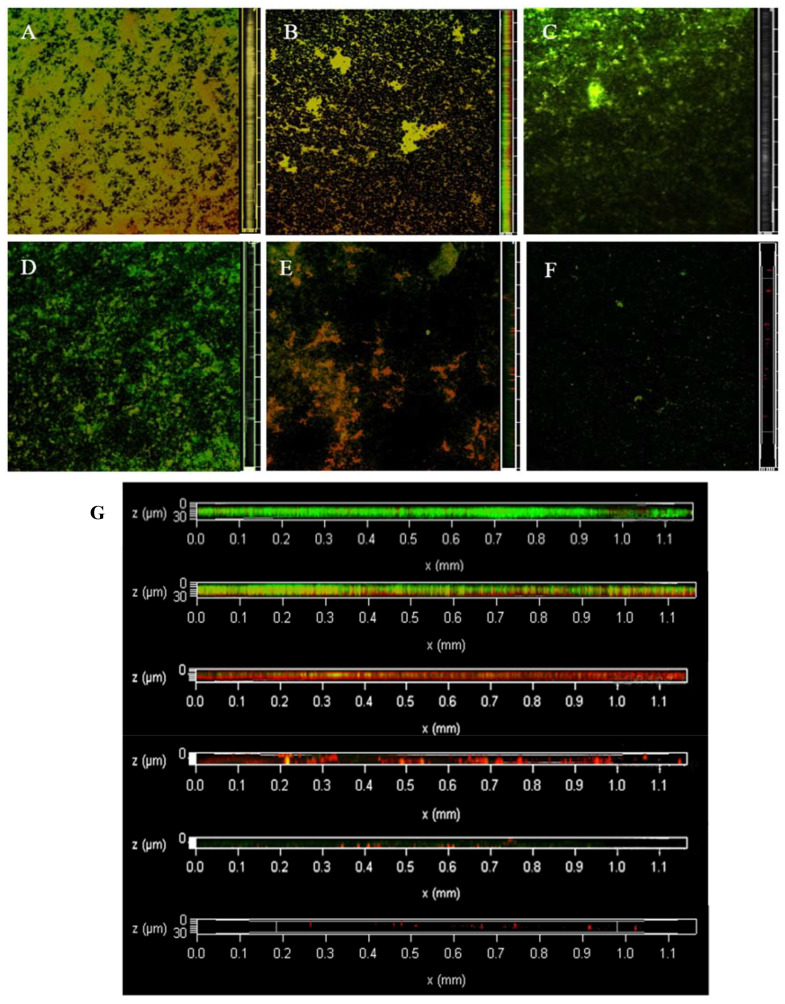
The changes of eDNA and eRNA in LM biofilm after NEHW treatment observed by CLSM; each sample randomly selects three visual fields. Images include front and side views of the biofilm under CLSM. Scale bar—100 µm. (**A**–**F**) represent treatment times of 0, 2, 4, 6, 8, and 10 min, respectively. (**G**) is an enlarged version of all the images on the right and is also a right-hand view of the biofilm, from top to bottom, for SAEW treatments 0, 2, 4, 6, 8 and 10 min respectively. NEHW was the abbreviation of non-electrolytic hypochlorite water.

**Figure 11:**
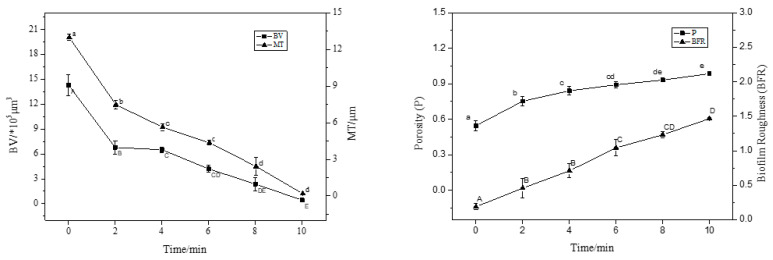
The CLSM images analysed by ISA-2 software to determine the biovolume (BV), mean thickness (MT), porosity (P) and biofilm roughness (BFR) of the biofilm. Data were expressed by mean measurements ± standard deviation (SD) and all treatments and determinations were performed in triplicate. The different letters indicate significant differences (*p* < 0.05).

Changes in the three-dimensional structure of LM during sterilisation with NEHW were observed using CLSM, and the resulting images were analysed using ISA-2 software. As shown in Figure 10 and Figure 11, the bacterial extracellular matrix decreased with an increase in NEHW action time, and the adhesion of bacteria to the carrier surface decreased [34]. The biofilm was gradually removed, the fluorescence gradually decreased, BV decreased by 8.91, 12.98, 16.63, 22.42, and 23.16 × 10^3^ μm^3^, while MT decreased by 8.43, 11.69, 14.33, 16.52, and 16.87 μm at 2–10 min, respectively, decreasing significantly compared to the control (*p* < 0.05), and BFR and P increased steadily.

### 3.8. Dyeing with AO after BF Treatment with AEW and ALEW

**Figure 12:**
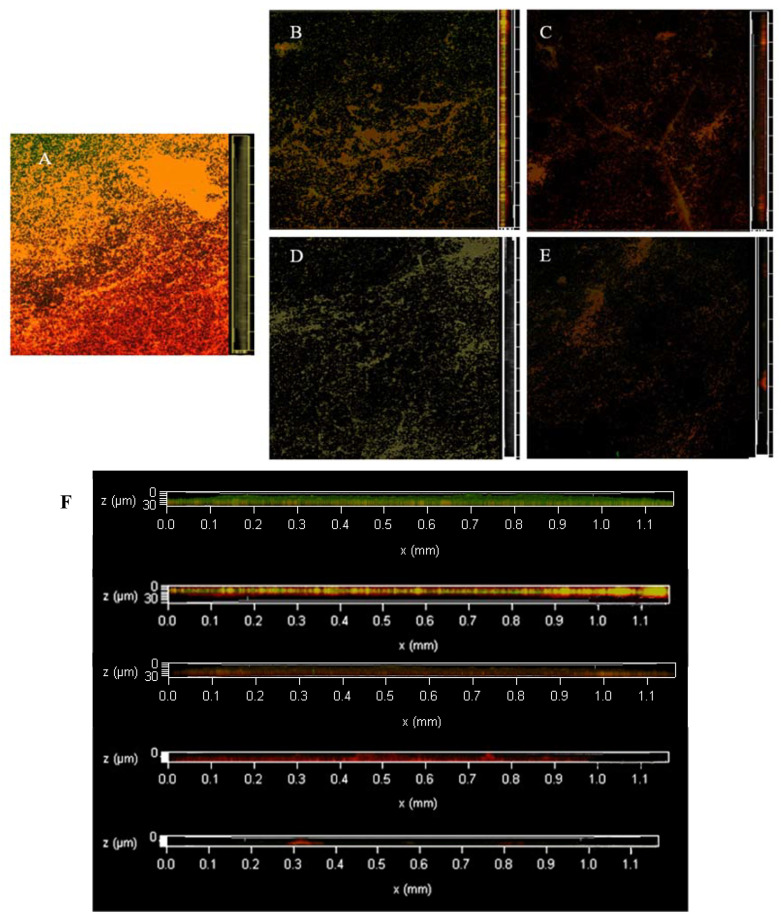
The changes of eDNA and eRNA in LM biofilm after AEW and ALEW treatment observed by CLSM; each sample randomly selects three visual fields. Images include front and side views of the biofilm under CLSM. Scale bar—100 µm. (**A**) is control, (**B**) is S_3_A_1_S_1_ treatment, (**C**) is A_1_S_4_ treatment, (**D**) is S_6_A_2_S_2_ treatment, and (**E**) is A_2_S_8_ treatment. (**F**) is an enlarged version of all the images on the right and is also a right-hand view of the biofilm, from top to bottom, for AEW and NEHW treatments 0, 2, 4, 6, 8 and 10 min respectively. AEW was acidic electrolytic water, and ALEW was alkaline electrolysed water.

**Figure 13:**
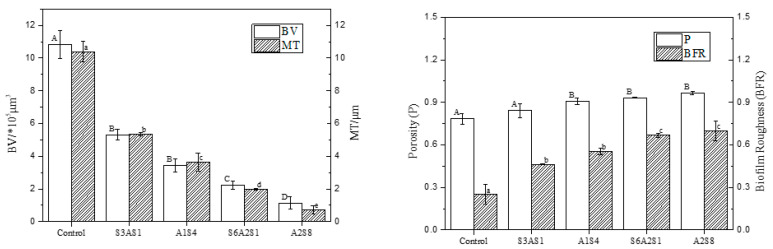
The CLSM images analysed by ISA-2 software to determine the biovolume (BV), mean thickness (MT), porosity (P) and biofilm roughness (BFR) of the biofilm. Data were expressed by mean measurements ± standard deviation (SD) and all treatments and determinations were performed in triplicate. The different letters indicate significant differences (*p* < 0.05).

Figure 12 and Figure 13 show that the eDNA and eRNA of the control (0 min) were evenly distributed, and the changes in each treatment showed that the eDNA and eRNA contents were gradually reduced. The extracellular eDNA and eRNA of the S_3_AS_1_ treatment were mostly removed; only a few gathered and were difficult to remove. On the other hand, the BFs in the A_1_S_4_, S_6_A_2_S_2_, and A_2_S_8_A treatment were gradually reduced, BV and MT were decreased, and P and BFR also increased steadily. The roughness increased because the BF no longer became flat and smooth after sterilisation. Instead, the grooves appeared more uneven, indicating that the elimination effect was more significant. The protective layer formed by the EPS was destroyed and the bacterium leaked out. However, the A_2_S_8_ treatment, with the most significant elimination effect, also had some extracellular polymers that had not been removed.

### 3.9. Multi-Mode Microplate Reader Validation Test

To further verify the changes in DNA and RNA contents in EPS, the results obtained from the multi-mode microplate reader were combined with the standard curve to derive the specific contents of extracellular DNA and RNA.

**Figure 14:**
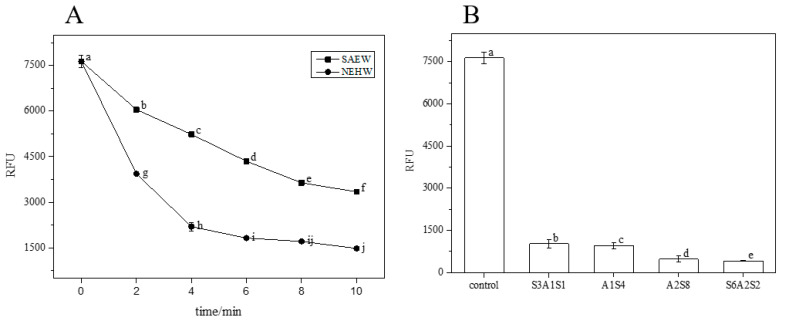
The RFU of residual eDNA content in each treatment. (**A**) is the RFU of residual eDNA after SAEW and NEHW treatment. (**B**) is the RFU of residual eDNA after AEW and ALEW treatment. The different letters indicate significant differences (*p* < 0.05). Data were expressed by mean measurements ± standard deviation (SD) and all treatments and determinations were performed in triplicate.

From Figure 14, both SAEW and NEHW treatment of LM showed a significant decrease in DNA content. Table 2 shows the eDNA content of each treatment. NEHW had a stronger ability to remove DNA than SAEW, and the difference was greatest at 4 min. After 10 min of treatment, the relative fluorescence intensity of SAEW was 3700 and the relative fluorescence intensity of NEHW was 1500. Both reached a relatively small level, thus verifying that the DNA was eluted from the BF.

**Figure 15:**
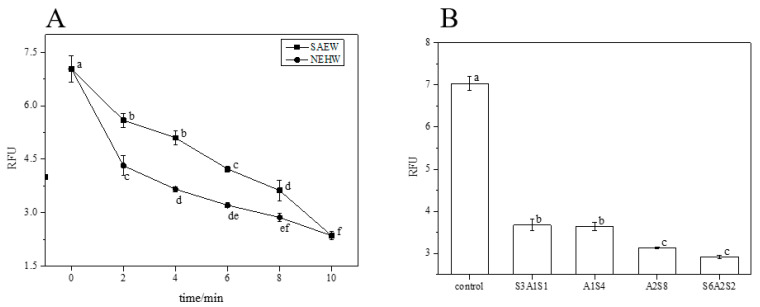
The RFU of residual eRNA content in each treatment group. (**A**) is the RFU of residual eRNA after SAEW and NEHW treatment. (**B**) is the RFU of residual eDNA after AEW and ALEW treatment. The different letters indicate significant differences (*p* < 0.05). Data were expressed by mean measurements ± standard deviation (SD) and all treatments and determinations were performed in triplicate.

Figure 15 and Table 3 show that the eRNA content of LM BFs treated with both SAEW and NEHW compared to the control group (0 min) was steadily reduced. NEHW had a stronger ability to remove eRNA than SAEW, with the largest difference observed at 4 min. After 10 min of treatment, the relative fluorescence intensity of both the slightly acidic and NEHW was 2.2, reaching a relatively small level. The fluorescence intensity of extracellular RNA after alternating acidic and alkaline electrolytic water was lower than that of the blank group, indicating that the eRNA in the BF was indeed removed.

### 3.10. Degradation Rate and Reaction Order of SAEW and NEHW Treated at Different Times

The data in Table 4 can be calculated based on Equations (1)–(3) and the remaining eDNA and eRNA content after treatment. As shown by R^2^, the degradation reaction of eDNA and eRNA in SAEW and NEHW is consistent with first-order kinetic reaction characteristics (R^2^ > 0.95). The degradation constants (K) were different for each treatment. eDNA was degraded at a higher rate in the NEHW treatment, which indicated that NEHW had a better removal effect on eDNA, whereas SAEW had a slightly better removal effect on eRNA. eDNA and eRNA are important components of the BF, indicating that the elimination of BF per unit time is directly proportional to the amount of BF. Therefore, the more BF there is, the more BF is removed per unit time.

## 4. Conclusions

In this study, three areas of biofilm structure were randomly selected to observe the interaction between biocides and biofilm in real time through confocal laser scanning electron microscopy. The fluorescence of different colours and intensities was used to observe the microstructure, breakage and changes in biofilm roughness of the biofilm, and to construct a three-dimensional structure of the biofilm based on the local density, volume, spatial location and distance of the fluorescence signals to summarise the changes of fungicides on the structure of LM biofilm on glass surfaces and to elucidate and verify the theory of layer-by-layer elimination of biofilm in *Listeria monocytogenes*. The fluorescence intensity was quantified using biological digital image processing software, Matlab and ISA software using information such as volumetric surface area ratio, porosity, average and maximum diffusion distance and surface area between voids, fractal dimensions, average run length (X, Y and Z directions), aspect ratio and based on digital biofilm images, The volume, biomass, average biofilm thickness, maximum biofilm thickness, bio-roughness and uniformity of LM biofilms were calculated. The results show that the LM biofilm decreases layer by layer with increasing sterilisation time, which proves the theory of layer-by-layer elimination of biofilm.

## 5. Discussion and Prospects

The experimental results showed that the BF of LM gradually became loose and thin from the initial density and tightness after the action of the three treatment, which is in accordance with the hypothesis of this study that the removal of LM BF is a dynamic layer-by-layer removal process. This study utilises a specialised high-resolution CLSM image acquisition method, which is one of the most widely used tools to study biofilm structure. The use of CLSM allows an invasive examination and subsequent computer reconstruction of mature BFs without significant distortion of the structure, resulting in an enhanced conceptual image of the microbial BF structure present in the situation. Furthermore, this approach allows the BF structure to be viewed from different angles, thereby identifying the general structure. Thus, laser confocal microscopy and computer image analysis can reveal BF morphology and more complex structures [35], enabling detailed 3D visualisation and assessment of biofilms. This is a tool that can be used to detect and visualise biofilm matrix components [36], which in turn provides detailed quantitative characterisation of the internal microstructure and enables the non-destructive study of BF structure using specific fluorescent markers and the ability to quantitatively characterise BFs in conjunction with image analysis software. The analysis software selected ISA-2 to calculate its biomass, and statistical analysis of the data showed that the BF became no longer flat and smooth after sterilisation but appeared in grooves. The biomass and BF thickness also decreased gradually with the extension of sterilisation time. The roughness of the measured extracellular nucleic acid was smaller, which is perhaps because the detected extracellular polymer was stickier and smoother, whereas the bacterium itself was rougher. Extracellular DNA (eDNA) and RNA (eRNA) are key components of EPS. It is released extracellularly through active bacterial secretion and cytolytic action, adsorbs to the bacterial cell surface, and extends outwards, thus promoting BF formation. In addition, it can provide nutrients for bacterial metabolism, act as a “gene store” for gene-level transmission, cross-link with extracellular proteins and polysaccharides, and maintain the BF spatial structure [37]. At the death of the bacterium and the destruction of the extracellular polymer structure, the adhesion of the BF to the carrier decreases, and the presence of eDNA promotes the adhesion of bacteria to other carrier surfaces to form biofilms. Therefore, the reduction of eDNA decreases the adhesion of bacteria on the surface of the attachment [38], and the destruction and removal of eDNA breaks the regulation of the population sensing system (QS). Therefore, biocides can have a better removal effect [39]. The more intense the means of biocide destruction, the lower the adhesion. Thus, the biofilm gradually becomes thinner and eDNA removal bacteria lose resistance, increasing the impact of biocides on the bacteria themselves [40]. The results of fluorescence zymography also show that extracellular nucleic acids are in a dynamic process of decline.

BFs are aggregates of microorganisms, in which cells are often embedded in self-generated extracellular polymeric matrices that adhere to each other and/or to surfaces [41]. Fungicides cause the physical release of cells by disrupting the chemical composition of the BF matrix, the disruption and degradation of which are major factors in BF dispersion [42]. Once the matrix is disrupted, BF cells separate as individual cells or larger cell clusters, leaving hollow microcolony structures that make it easier for the biocide to reach the bacteria themselves. The hydroxyl radicals contained in several of the biocides used herein have potential interactions with key biomolecular biofilm components, including alkanes, alcohols, carboxylic acids, and amines, and organic molecules containing hydroxyl and carboxyl groups may act as trapping agents for OH radicals in BFs. In addition, OH radicals may lead to hydrogen abstraction and subsequent molecular damage, and the interaction of hydroxyl radicals with biomolecules (polysaccharides, lipids, and proteins) in BFs leads to the formation of biomolecular radicals that initiate structural damage in BFs [43]. Electrolytic and NEHW hold great promise as a biocide for removing BFs from natural and natural surfaces, making it suitable for application in all environments that would be contaminated with microorganisms, including the food and medical industries. As discussed in this article, laboratory tests have shown a high reduction in colony-forming units under a range of conditions. However, in an amplified industrial environment, factors that may affect the efficacy of a biocide may be interactions with other components of water, such as pesticide residues and heavy metals. These interactions may alter the composition and efficacy of biocides [44]. In addition, physicochemical factors such as temperature and pH also play a role in making the biocide less effective than it could.

BF formation is a complex dynamic process, and cell-vector attachment is one of the most important processes, representing a turning point from planktonic BFs to BF life patterns [45]. The combination of molecular biology and microbiology can assess BF biomass and viability using the physical or chemical properties of BF dynamics [46]. Various microscopic methods assess BF properties in a more direct way and can show the connection between BF spatial organisation and BF function [47,48]. Currently, research is limited to a single strain. In contrast, in nature, microorganisms are often present in multi-strain mixtures, and the study of these isolated strains can shed some light on how they differ from the mixed strains that exist in their natural state. BF behaviour and resistance are also influenced by environmental factors as well as the complexity of EPS matrices, which interact with each other and establish cooperative or competitive behaviours that lead to increased or decreased bacterial resistance. Mixed BF consortia have been found to contaminate food and water surfaces and are often highly resistant to sterilisation treatments. For example, pathogens such as LM and *Salmonella typhimurium* can exist as BF communities and cross-contaminate food surfaces [49]. eDNA has shown strong protection against stress from metal ions, oxidising agents, and attacks induced by the host immune system. The world of microorganisms is a complex environment, and the interaction and information exchange between various microorganisms leads to the elimination of BFs layer-by-layer, which cannot be explained by a single-strain environment. The volume of BFs in late growth is larger than that of BFs in early growth, which may indicate that the size and number of microcolonies increase over time as the BF ages. BF formation requires cell adhesion, surface treatment, and EPS production, all of which are energy-consuming processes. However, this is evolutionarily plausible considering the enormous benefits of EPS protection for bacteria under nutrient-deprived conditions [50]. In combination with the experimental results, the colonies in the low-density regions of EPS may indicate some degree of developmental structural organisation, and the degree of disruption of the BF structural organisation during sterilisation was also shown. It can be hypothesised that these thin BFs are immature and no longer intact. With continued sterilisation treatment, it is apparent that the BF can be consistently reduced in size and disrupted in function. These new findings help shape our understanding of how bacteria persist and resist the environment after using nutrients to generate mature biofilms, and they can provide support for limiting and controlling bacteria.

*L. monocytogenes* represents a serious concern for human health due to its ability to colonise food processing environments, including equipment used in food processing operations. *L. monocytogenes* adheres to a variety of surfaces, including stainless steel, polystyrene and glass, and it is capable of forming biofilms [51]. Therefore, the next step in the research programme is to build on previous studies by culturing *Listeria monocytogenes* on materials commonly used in industrial equipment, such as stainless steel, wood and polypropylene, to quantify the amount of LM biofilm production, extracellular polymer content, LM permeability and damage, and to quantitatively analyse the physiological and biochemical parameters of the biofilm during clearance. We further verify that the removal of LM biofilm conforms to the theory of layer-by-layer elimination of biofilm in *Listeria monocytogenes*.

The results of this study demonstrate that the removal process of *Listeria monocytogenes* biofilm on the surface of glass carriers is layer by layer, and to achieve better biofilm removal on the surface of production equipment, similar biocides as in this paper can be used to elute the biofilm not only to inactivate bacteria but also to remove the biofilm layer by layer. We will continue to investigate whether LM biofilm on other surfaces of food production lines of different materials is also consistent with this removal theory to provide a theoretical basis for further investigation into the removal mechanism of LM biofilm. We hope to provide researchers with a clearer visualisation of the dynamic removal process so that they can better understand the structural changes in the biofilm as it is removed, use more effective means to prevent biofilm formation, facilitate the research and use of other biocides or antimicrobial agents, and establish new modes of action to improve their utilisation. The process of developing new methods for a more efficient and environmentally friendly prevention and control of *Listeria monocytogenes* contamination in the food industry, and adapting them more quickly to the production chain to improve business efficiency, is important for the advancement of the industry.

## Figures and Tables

**Table 1:** The fluorescent dye that binds tightly to biofilm.

Fluorescent Dye	Binding Substances	Excitation Wavelength/nm	Emission Wavelength/nm
PI	Dead cells	485	630
Syto^®^9	live cells	485	530
AO	DNA	490	528
AO	RNA	555	620

PI was the abbreviation of propidium iodide, and AO was the abbreviation of acridine orange.

**Table 2:** The residual eDNA content in the biofilm of *Listeria monocytogenes* in each treatment.

	SAEW	NEHW	A_1_S_4_	A_2_S_8_	S_3_A_1_S_1_	S_6_A_2_S_2_
Control	967.76 ± 3.25	967.76 ± 3.25	967.76 ± 3.25	967.76 ± 3.25	967.76 ± 3.25	967.76 ± 3.25
2 min	102.64 ± 2.01	45.18 ± 1.33				
4 min	32.51 ± 0.51	10.45 ± 0.12				
5 min			0.085 ± 0.013		0.077 ± 0.011	
6 min	9.31 ± 0.12	2.26 ± 0.07				
8 min	3.40 ± 0.0044	0.22 ± 0.0021				
10 min	2.24 ± 0.0084	0.16 ± 0.0023		0.039 ± 0.0093		0.036 ± 0.0032

unit: μg/mL; Values were obtained by three replicated measurements; SAEW was the abbreviation of slightly acidic electrolytic water, and NEHW was the abbreviation of non-electrolytic hypochlorite water. In A_1_S_4_, A_2_S_8_, S_3_A_1_S_1_, and S_6_A_2_S_2_ treatment, S stands for strongly acidic electrolytic water, A stands for strongly alkaline electrolytic water, and the number represents the action time.

**Table 3:** The residual eRNA content in the biofilm of *Listeria monocytogenes* in each treatment.

	0 min	2 min	4 min	6 min	8 min	10 min
SAEW	60.694 ± 0.23	4.842 ± 0.11	3.228 ± 0.024	2.201 ± 0.022	1.198 ± 0.017	1.079 ± 0.013
NEHW	60.694 ± 0.23	4.078 ± 0.12	2.559 ± 0.013	1.789 ± 0.067	1.132 ± 0.015	0.967 ± 0.032

unit: μg/mL; Values were obtained by three replicated measurements; SAEW was the abbreviation of slightly acidic electrolytic water, and NEHW was the abbreviation of non-electrolytic hypochlorite water. A_1_S_4_, A_2_S_8_, S_3_A_1_S_1_, and S_6_A_2_S_2_ (where S stands for strongly acidic electrolytic water, A stands for strongly alkaline electrolytic water, and the number represents the action time).

**Table 4:** Degradation rate and reaction order of SAEW and NEHW treated at different times.

		Zero-Order Reaction		First-Order Reaction		Second-Order Reaction	
		K_0_/min^−1^	R^2^	K_1_/min^−1^	R^2^	K_2_/min^−1^	R^2^
eDNA	SAEW	11.3955	0.6374	0.4954	0.9661	0.0431	0.8778
	NEHW	5.0135	0.5746	0.7574	0.9567	0.8453	0.7829
eRNA	SAEW	0.4778	0.9068	0.1997	0.9619	0.0983	0.9297
	NEHW	0.3825	0.8779	0.1847	0.9731	0.1035	0.9729

SAEW was the abbreviation of slightly acidic electrolytic water, and NEHW was the abbreviation of non-electrolytic hypochlorite water.

## Data Availability

Data is contained within the article.
